# Rational Fabrication of Ionic Covalent Organic Frameworks for Chemical Analysis Applications

**DOI:** 10.3390/bios13060636

**Published:** 2023-06-08

**Authors:** Jing Yu, Liuna Luo, Hong Shang, Bing Sun

**Affiliations:** School of Science, China University of Geosciences (Beijing), Beijing 100083, China; 2119210047@email.cugb.edu.cn (J.Y.); 2019220035@email.cugb.edu.cn (L.L.); shanghong@cugb.edu.cn (H.S.)

**Keywords:** biosensing, chemical analysis, enrichment, ionic covalent organic frameworks, ion sensing, structural design

## Abstract

The rapid development of advanced material science boosts novel chemical analytical technologies for effective pretreatment and sensitive sensing applications in the fields of environmental monitoring, food security, biomedicines, and human health. Ionic covalent organic frameworks (iCOFs) emerge as a class of covalent organic frameworks (COFs) with electrically charged frames or pores as well as predesigned molecular and topological structures, large specific surface area, high crystallinity, and good stability. Benefiting from the pore size interception effect, electrostatic interaction, ion exchange, and recognizing group load, iCOFs exhibit the promising ability to extract specific analytes and enrich trace substances from samples for accurate analysis. On the other hand, the stimuli response of iCOFs and their composites to electrochemical, electric, or photo-irradiating sources endows them as potential transducers for biosensing, environmental analysis, surroundings monitoring, etc. In this review, we summarized the typical construction of iCOFs and focused on their rational structure design for analytical extraction/enrichment and sensing applications in recent years. The important role of iCOFs in the chemical analysis was fully highlighted. Finally, the opportunities and challenges of iCOF-based analytical technologies were also discussed, which may be beneficial to provide a solid foundation for further design and application of iCOFs.

## 1. Introduction

Environment sustainability, food security, and life healthy have drawn more and more attention in modern society, which is correlated with the resolutions to address the challenges in economic development and social progress. Exactly recognizing and rapidly removing the deleterious substances or pollutants in the environment, food, water, and organisms provide effective ways to govern the potential risks resulting from these matters [1,2,3]. Moreover, it is also desirable to monitor the ambient condition changes of the surroundings. However, the complexity of the natural samples usually requires complicated pretreatment, accurate recognition, specific detection, and acceptable reliability in the presence of unpredicted interferences. On the other hand, the trace analytes in the surroundings also call for an efficient enrichment procedure and lower detection of limits to the analytical protocols. Therefore, it is still an important topic to conveniently, rapidly, and sensitively recognize and detect the target analytes avoiding sophisticated and time-consuming procedures.

The development of materials science, nanotechnology, and bionic design provides various functionalized materials that promise extraction efficiency and sensing performance for advanced chemical analytical methods, which boosts the increasing analytical research and extensive detecting applications in the fields of food security, environment monitoring, life-health, etc. [4,5,6]. Recently, porous materials, including inorganic porous materials, organic-inorganic porous materials, and organic porous materials have played an important role in analytical testing and biosensing with predesigned structures and versatile physiochemical properties [7,8]. Among them, inorganic porous materials such as zeolite, mesoporous oxides, and porous carbon can effectively extract and analyze target analytes benefiting from their high specific surface area, effective transducing performance, low cost, and good stability [9,10,11,12,13,14]. Compared to inorganic porous materials, organic porous materials, particularly metal-organic frameworks (MOFs), covalent organic frameworks (COFs), hydrogen-bonded organic frameworks (HOF), and porous aromatic frameworks (PAFs), represent predesigned molecular structures, higher specific surface area, predictable permanent pore channels, uniformly distributed active center, and tunable functionalities, which make them more advantageous applied in analytical fields [15,16,17,18,19,20].

COFs are a class of rapidly developed crystalline porous organic materials formed by strong organic covalent bonds of small organic molecules containing light elements (C, H, N, O, etc.) via structural design and optimized synthetic methodologies, and have increasingly attracted extensive attention by realizing multi-function and multi-scene applications [21,22]. In this context, ionic covalent organic frameworks (iCOFs) emerge as crystalline organic polymers composed of ionic frameworks and corresponding counter-ions [23,24]. By combining the advantages of porous ionic polymers (PiPs) and COFs, iCOFs not only retain the design concept of COFs with unique atomic periodicity and backbone porosity but also involve groups with opposite charges in the skeletons of iCOFs through electrostatic interactions or ion exchange [25]. These features benefit the wide applications of iCOFs in ion conduction, catalysis, energy storage, adsorption, and separation [26,27,28,29,30,31,32]. More intriguingly, the permanent porosity, precisely designable structure, and unique ionic sites endow iCOFs as good candidates for effective and selective extraction of tracing elements and matters. On the other hand, the physicochemical properties with high responsiveness to electricity, electrochemistry, and photo-irradiation can be achieved by modulating the structures and charges of iCOF, making them potential transducers to rapidly and sensitively detect the target analytes [33]. Therefore, iCOFs are promising to be applied in analytical fields with a wide range of application prospects including but not limited to the rapid detection and treatment in environmental and food fields and biosensing to specific biomolecules.

At present, most of the domestic and international reviews provide a relatively comprehensive summary of the synthetic strategies, ion conduction, and catalytic applications of iCOFs, but there are rare summaries and analyses of iCOF in analytical applications. In this review, we primarily summarized the typical fabrication strategies of iCOFs for analytical pretreatment and sensing applications following the Introduction part. Then, the applications of iCOFs in enrichment and extraction, biosensing, molecules detection, ion sensing, humidity/temperature monitoring, etc. are mainly highlighted. Finally, the potential challenges of iCOFs for analytical applications are also discussed, which may contribute to the advanced progress of iCOF-based analytical methodologies.

## 2. Structural Design of iCOFs for Analytical Application

**Figure 1:**
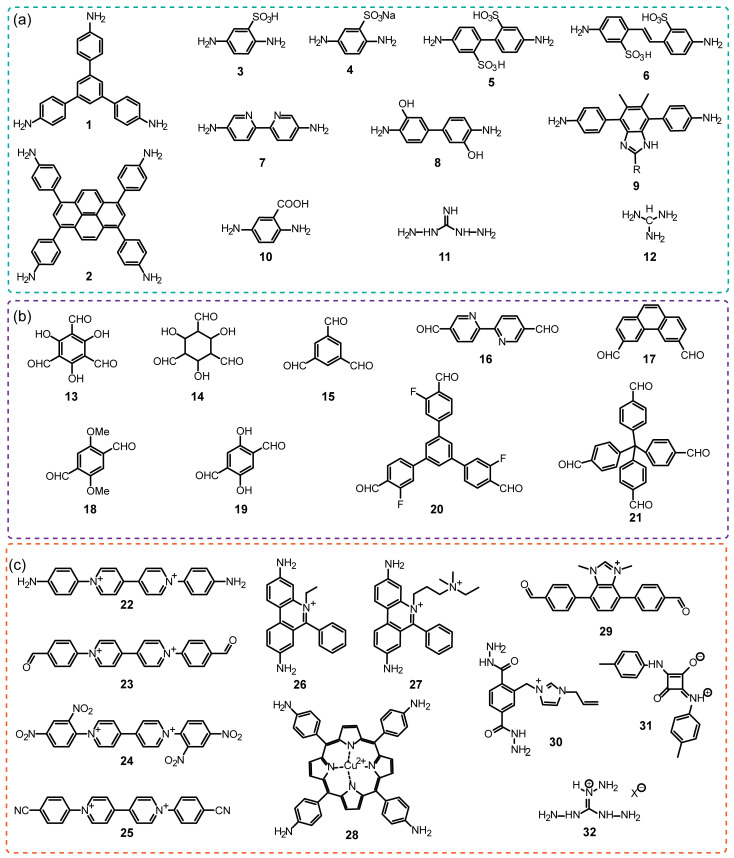
Typical building blocks used for iCOFs construction mentioned in this review: (**a**) neutral amine monomers (**1**–**12**), (**b**) neutral aldehyde monomers (**13**–**21**) and (**c**) typical charged building blocks (**22**–**30** and **32**) and linkages (**31**).

**Figure 2:**
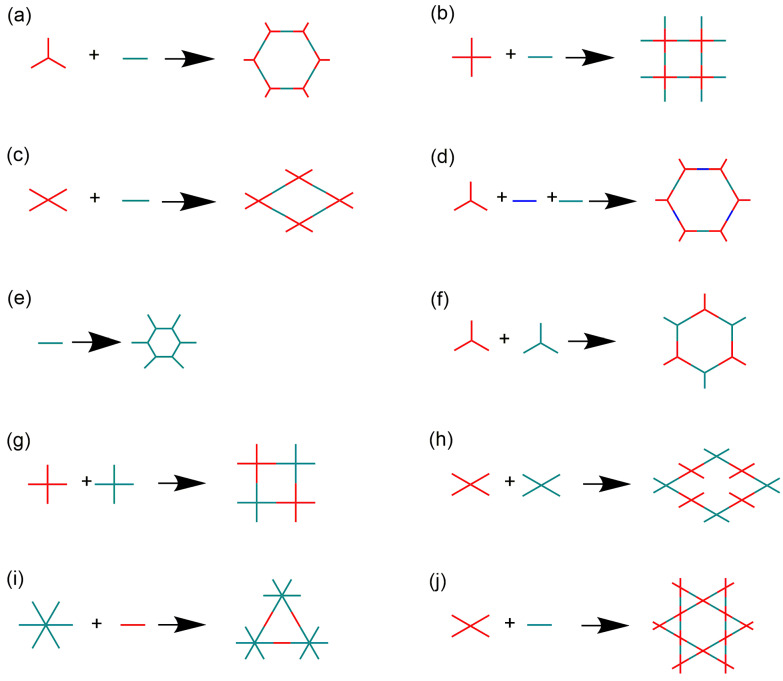
(**a**–**j**) Different topologies of 2D iCOFs based on the molecular structures of building blocks to construct iCOFs summarized in the present work.

The iCOFs are usually fabricated by using the direct synthetic strategy and post-synthesis modification. The direct synthesis is typically performed based on the polycondensation from the tunable ionic monomer molecules and charge distribution control. Typical ionic building blocks such as ethidium bromide (EB), 2,5-diaminobenzenesulfonic acid, and so on (as shown in Figure 1) have been developed for constructing highly crystalline iCOFs linked with non-ionic monomers [34,35,36]. Non-ionic monomers are also used to construct ionic COFs by forming spiroborate ester bonds, square acid cyanine bonds, and other bonds [37]. Based on the condensation of building blocks with predesigned symmetry and conformation (as shown in Figure 2), two-dimensional (2D) iCOF structures with typical overlapping stacks or 3D iCOFs with multiple interspersed structures can be obtained. On the other hand, post-modification of the well-defined COF porous structures is another effective way to construct the functionalized iCOFs with controllable charge located on the pore walls [38]. The post-modification method to introduce ionic groups into COFs also results in good controllability in the crystallization and ionization properties of iCOFs.

**Figure 3:**
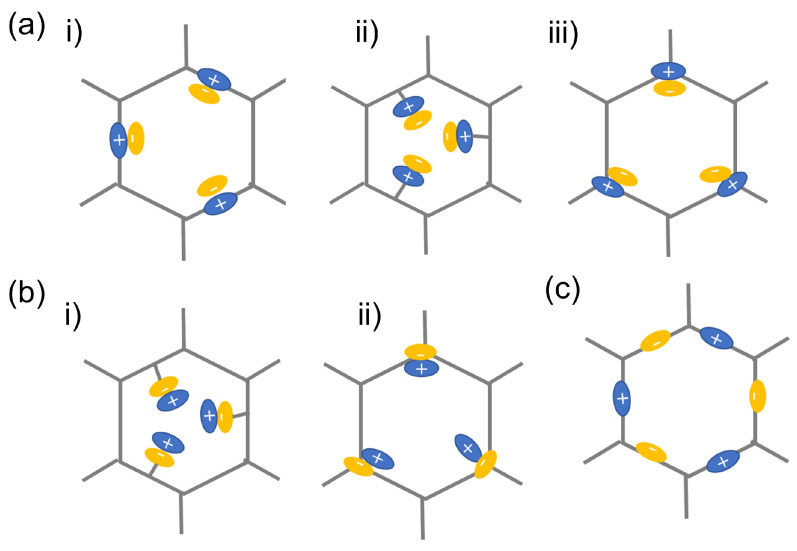
Schematic illustration on the typical classification of iCOFs: (**a**) cationic COFs with Type i, Type ii, and Type iii, respectively; (**b**) anionic COFs with Type i and ii, respectively; (**c**) zwitterionic COFs. Reproduced with permission from [23]. Copyright 2021 Elsevier.

Through equilibrium electrostatic interactions, the ions are either on the frameworks or on the side chains. According to the different charge distributions, iCOFs can be divided into cationic COFs, anionic COFs, and zwitterionic COFs (Figure 3), which demonstrate promising applications in chemical analysis fields. The typical iCOFs and their structural characteristics are summarized in Table 1.

### 2.1. Cationic COFs

Cationic COFs are the iCOFs holding positive charges. The cationic portion can be located on frameworks by pre-modifying ionic motifs in linking/node monomers or distributed on side chains via the post-synthesis modification (Figure 3a).

**Figure 4:**
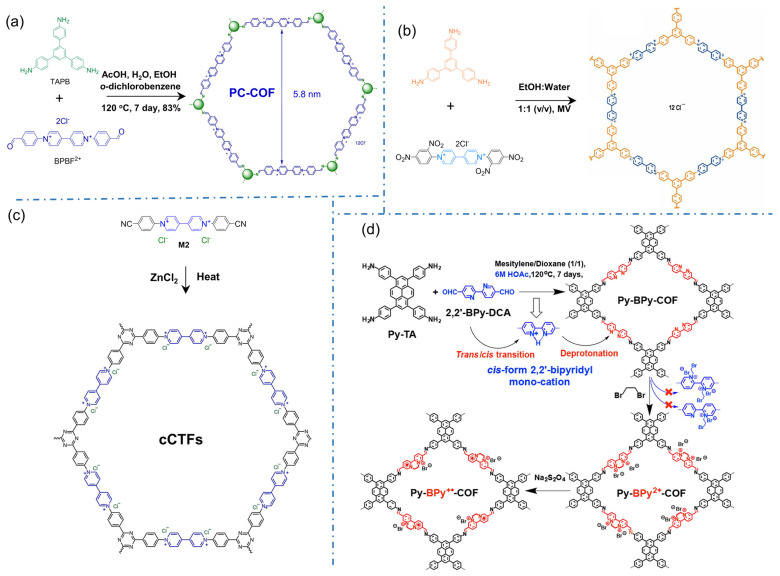
Typical cationic COF structures based on bipyridinium building blocks: (**a**) 2D honeycomb-styled PC-COF. Reprinted with permission from [35]. Copyright 2016 Royal Society of Chemistry. (**b**) Viologen-based COFs. Reprinted with permission from [61]. Copyright 2017 American Chemical Society. (**c**) Charged covalent triazine frameworks (cCTFs). Reprinted with permission from [39]. Copyright 2017 American Chemical Society. (**d**) Py-BPy^2+^-COF. Reprinted with permission from [49]. Copyright 2019 American Chemical Society.

Viologens are the typical cationic motifs with bipyridinium structures for directly constructing iCOFs. In 2016, Li’s group reported the first case of polycation 2D COFs (denoted as PC-COF) by Schiff-base reaction with 1,3,5-tri(4-aminophenyl)benzene (TAPB) and 1,1′-bis(4-formylphenyl)-4,4′-bipyridine dichloride (BFBP^2+^⸱2Cl^−^) [35], in which the cations PF_6_^−^ and Cl^−^ can be installed as counterions (Figure 4a). Powder X-ray diffraction (PXRD) and small angle X-ray scattering (SAXS) experiments indicated its 3D porous frames stacked from 2D layers. The adjacent 4,4′-bipyridinium (BIPY) dications were stacked in an eclipsed pattern with chloride counterions being sandwiched in between the layers. Due to its high stability, the 3D polycationic COF is expected to be a promising adsorbent for the enrichment of target analytes and the detection of trace substances. The iCOFs containing viologens were also synthesized through the Zincke reaction by Trabolsi’s group (Figure 4b) [61]. In 2017, Buyukcakir et al designed and synthesized the first charged covalent triazine framework (cCTF, Figure 4c) using cyanophenyl-substituted viologens as building blocks [39]. The surface area and pore volume/size of cCTFs can be simply controlled by varying the synthesis temperature and the ZnCl_2_ content. In addition to the high surface area and thermal/chemical stability, the electrostatic interaction between the target molecules and the charge center of the bipyridinium unit makes it one of the promising candidates for the enrichment and detection of the target analyte.

Similar to bipyridinium structures, bipyridine in the framework of neutral COFs is regarded as a metal-binding site that can be ionized by introducing metal or proton ions [48]. In 2019, Mi et al. transformed COF based on 2,2′-bipyridine from neutral to positively charged skeleton with high crystallinity (Py-BPy^2+^-COF, Figure 4d) [49]. The formation of acid-catalyzed COF enables *cis*-configuration of the 2,2′-bipyridine portions whose stability comes from the stacking of two-dimensional layered structures. More impressively, the cationic radical COF exhibits a significant structurally enhanced photothermal conversion due to the extended exchange transfer between π-coupled intermediate layers.

**Figure 5:**
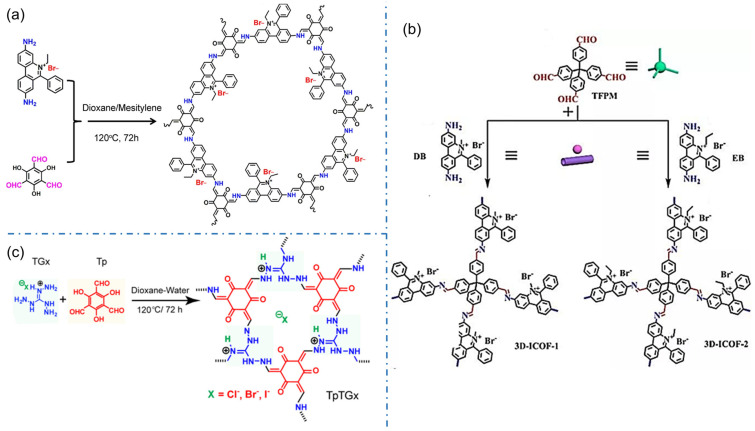
Typical EB-based and TG-based cationic COF structures. (**a**) EB-COF:X (X=F, Cl, Br, I). Reprinted with permission from [34]. Copyright 2016 American Chemical Society. (**b**) 3D-Ionic-COFs. Reprinted with permission from [25]. Copyright 2017 American Chemical Society. (**c**) TpTGx COF. Reprinted with permission from [40]. Copyright 2016 American Chemical Society.

The iCOFs based on ethidium bromide (EB, 3,8-diamino-5-ethyl-6-phenylphenanthrene bromide) are another typical cationic COFs with higher crystallinity. In 2016, Ma et al. designed and synthesized the cationic COF with high thermal and chemical stability by combining EB with 1,3, 5-triformylphlotrichol (TFP) in a Schiff-base reaction [34]. A series of charged cationic COFs (EB-COF:X, X=F, Cl, Br, I) were prepared for the first time by the ion exchange process (Figure 5a). The results show that the porosity and pore size of iCOFs can be finely regulated at the nanometer scale. The ion exchange process of EB-based-iCOFs also produces iCOF-based composites. In 2021, Jiang et al. designed and synthesized novel-magnetic cationic COFs (Fe_3_O_4_@EB-iCOFs) [62]. The results of X-ray diffraction, N_2_ adsorption-desorption analysis, and magnetic measurement demonstrated their good ionic properties, a large specific surface area, and strong magnetic responsiveness. The cationic EB monomer can also be used to construct 3D iCOFs. In 2017, Qiu’s group reported two kinds of highly crystalline 3D cationic COFs with a triple penetration diamond topology (3D-iCOF-1 and 3D-iCOF-2, Figure 5b) by condensing tetra(4-formyl phenyl)methane (TFPM) with two kinds of ionic chain segments 3,8-diamino-5-methyl-6-phenyl phenyl onium salt (DB) and 3,8-diamino-5-ethyl-6-phenyl phenyl onium salt (EB) [25]. The charge repulsion may change the penetration from the nine-fold penetration of the non-ionic COF-320 to the triple penetration. These 3D cationic COFs revealed excellent performance in size-selective ion exchange.

Besides, triaminoguanidine (TG) hydrochloride, a unique 3-linked ionic monomer, is also used to construct node-cationic COFs. In 2016, Banerjee et al. performed a pioneering study to synthesize a self-striping guanidine-based iCON (Figure 5c) using triuronic phloroglucinol (TP) and diaminoguanidine hydrochloride as monomers [40]. The iCONs with several thin layers have a good antibacterial effect against *Staphylococcus aureus* (*S. aureus*) and *Escherichia coli* (*E. coli*). Along the same line, diaminoguanidine hydrochloride can also react with benzene-1,3,5-triclocarban aldehydes, 2,5-dihydroxy terephthalic aldehydes, and other aldehydes to form cationic 2D COF with *hcb* topology [42]. In 2022, Das et al proposed a luminescent iCOF (TGH^+^⸱PD) composed of TG and phenanthroline fractions for the rapid detection of ammonia and primary aliphatic amines [43]. The deprotonation of the guanidine part by the amine limits the intramolecular charge transfer (ICT) and exhibits a strong enhancement of fluorescence emission. In addition, the presence of ordered pore walls allows for size selectivity between analyte molecules. The ionic fraction uptake of the guest via iCOFs leads to a significant change in luminescence, making them excellent candidates for chemical sensors.

### 2.2. Anionic COFs

Anionic COFs have an electronegative framework constructed from anionic monomers or connectors. The anionic portion can be pre-modified or post-modified on the side chains, and nodes of the frameworks to produce anionic COFs (Figure 3b).

**Figure 6:**
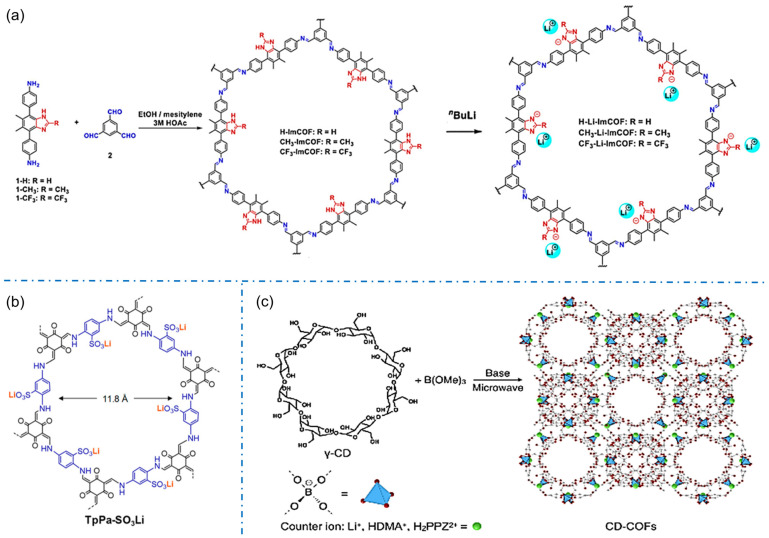
Typical anionic COF structures condensing from their corresponding building blocks: (**a**) imidazole iCOFs. Reprinted with permission from [57]. Copyright 2019 American Chemical Society. (**b**) TpPa-SO_3_Li. Reprinted with permission from [36]. Copyright 2019 American Chemical Society. (**c**) CD-COFs. Reprinted with permission from [58]. Copyright 2017 Wiley-VCH.

Imidazole functional groups tend to be deprotonated as anions, which are typical candidates for constructing anionic COFs. In 2019, Zhang’s group synthesized a series of crystalline imidazolium-ester COFs and obtained anionic COFs containing imidazolium-ester after lithium (Figure 6a), which were the first series of crystalline imidazolium-ester iCOFs applied to single-ion conductive COFs [57].

The development of sulfonyl construction iCOFs is more extensive for constructing anionic COFs. The iCOFs based on sulfonated acids (TFP-DABA) were first synthesized by Zhao’s group with a typical solvothermal method [63]. Subsequently, Yan et al. synthesized an β-ketonimine-linked functionalized spherical COF with sulfonic acid (TFP-BDSA COF) [64]. These COFs can realize the separation of anionic and cationic dyes based on a charge-dependent mode. In 2019, Lee et al. synthesized TpPa-SO_3_-COF by using 2,5-diaminobenzene sulfonic acid as a monomer with the sulfonic groups on its side [36]. The obtained TpPa-SO_3_Li containing free lithium ions and lithium-ion-anchored anions (Figure 6b) shows excellent conductivity of single lithium-ion. In 2020, Ma’s group proposed a 2D sulfonate anionic COF membrane with accessible ionic units and uniform nanochannels for the detection of cationic organic pollutants [54]. The anionic COF membranes have a permanent microporous structure in various solvents, and their charged nature allows the adsorption and enrichment of organic pollutants through charge control and thus the detection of that contaminants. In 2021, Hou et al. directly synthesized a self-supported TpPa-SO_3_H-COF membrane by interfacial method [65]. The TpPa-SO_3_H-COF membrane has a good potential in salt difference energy conversion due to its penetrating pores and high charge density.

The novel ionic structures also contribute to the design and fabrication of more iCOFs. In 2017, Zhang et al. first reported the design and synthesis of 3D COFs connected with spiroborate by the aliphatic group, γ-cyclodextrin (γ-CD), as well as a unique tetrahedral structural bond (spiroborate) through a simple microwave-assisted method (Figure 6c) [58]. These anionic COFs have a variety of balanced cations, such as Li^+^, HDMA^+^, H_2_PPZ^2+^, etc. The interaction between CD-COF and CO_2_ molecules can be effectively regulated by further changing the counterions in the pores. The existence of CDs makes crystalline porous solid materials more broad prospects in energy, biology, and environmental applications.

### 2.3. Zwitterionic COFs

**Figure 7:**
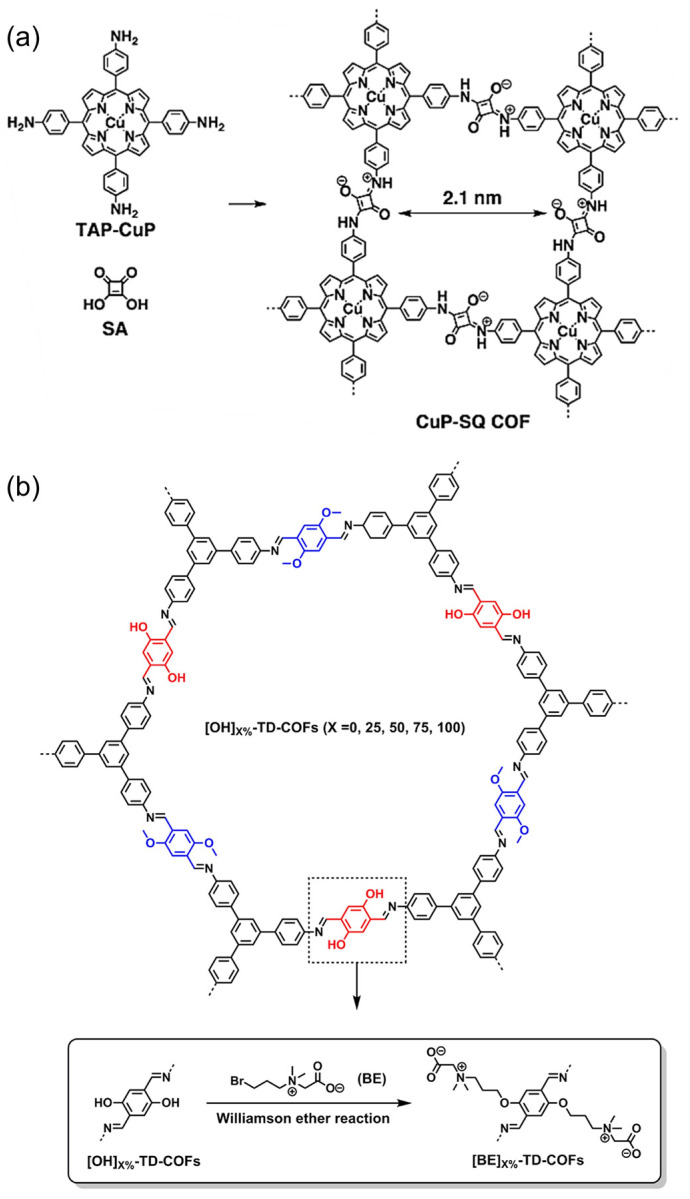
(**a**) Zwitterionic CuP-SQ-COF directly condensing from the corresponding building blocks. Reprinted with permission from [59]. Copyright 2013 Wiley-VCH. (**b**) Construction of zwitterionic [BE]_X%_-TD-COFs by post-modification. Reprinted with permission from [38]. Copyright 2018 American Chemical Society.

Zwitterionic COFs materials are mainly COFs with zwitterionic bonds on the frameworks and COFs modified by zwitterionic liquid (Figure 3c). In 2013, Jiang’s group synthesized CuP-SQ-COF connected with square-acid cyanine by bottom-up method (Figure 7a) [59]. Although there are a lot of positive and negative charges distributed in the frameworks, the material still showed good crystallinity and high specific surface area and enabled to induce singlet oxygen generation under visible light. A few years later, Mu et al. reported a class of new zwitterionic COFs ([BE]_X%_-TD-COFs, Figure 7b) [38], which was prepared by introducing beet base groups (BE) into the pre-synthesized frameworks through the pore surface engineering. The adjustable density of the BE groups, as well as the good crystallinity and porosity, give [BE]_X%_-TD-COFs a highly ordered distribution of binding sites.

**Figure 8:**
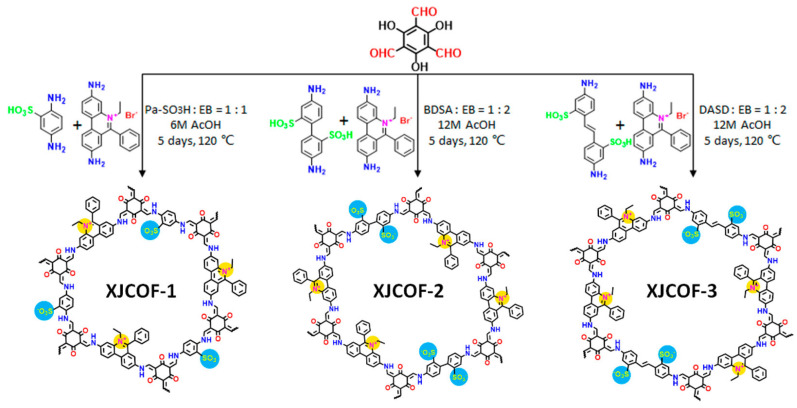
Construction of zwitterionic XJCOF-1, XJCOF-2, and XJCOF-3 by the mixed condensation of two amine monomers with Tp. Reprinted with permission from [60]. Copyright 2021 American Chemical Society.

Although only a few works were reported, the newly emerged zwitterionic COFs have drawn attention in the fields of medicine and proton transport. A novel magnetic zwitterionic COF, namely glutathione-functionalized thioether COF-based composite (Fe_3_O_4_@Thio-COF@Au@GSH) was designed and reported with fast magnetic responsiveness, regular porosity, and suitable surface area [66]. In 2021, Fu et al. designed and synthesized three kinds of zwitterionic COFs (Figure 8) [60]. Upon loading with triazole/imidazole, the amphoteric ionic groups in the COFs channels can induce complete deprotonation of proton carriers, producing more freely migrating protons.

The predesigned porous structures, abundant charge distribution, and tunable functionalities of iCOFs provide advanced materials for their applications in chemical analysis including extraction/enrichment, biosensing, ion-sensing, molecule detection, and the surroundings monitoring, which would be comprehensively discussed in the following part.

## 3. Applications of iCOFs in Chemical Analysis

### 3.1. Enrichment and Extraction

The high crystallinity, permanent pore structures, high specific surface area, and tunable charges endow iCOFs as good candidates to extract small molecules and enrich trace substances, contributing to achieving precision medicine, environmental protection, food safety, and so on. The iCOF characteristics for analytical methodology can be combined with new technologies as an effective way to improve sensitivity and accuracy, which were summarized in Table 2. Ma et al. prepared a petal-type COF composite (PS-IL-COF) decorated with ionic liquids [67]. The PS-IL-COF was used as adsorbents combined with liquid chromatography-tandem quadrupole mass spectrometry (LC-MS/MS) for the determination of allantoin in human plasma, which can be used for clinical studies. Under optimal conditions, the detection limits of PS-IL-COFs particles were 0.18 μg/L, 0.15 μg/L, and 0.016 μg/L for propofol, ketamine, and etomidate, respectively. Similarly, the Fe_3_O_4_@EB-TFB-iCOF composites were designed and synthesized by Lin’s group [68]. The extracted organic acid content by using Fe_3_O_4_@EB-TFB-iCOF composites was determined using high-performance liquid chromatography-UV analysis (HPLC-UV) with lower detection limits (0.1~0.49 ng/mL), indicating the possibility of highly sensitive detection of polar organic acids in the environment.

**Figure 9:**
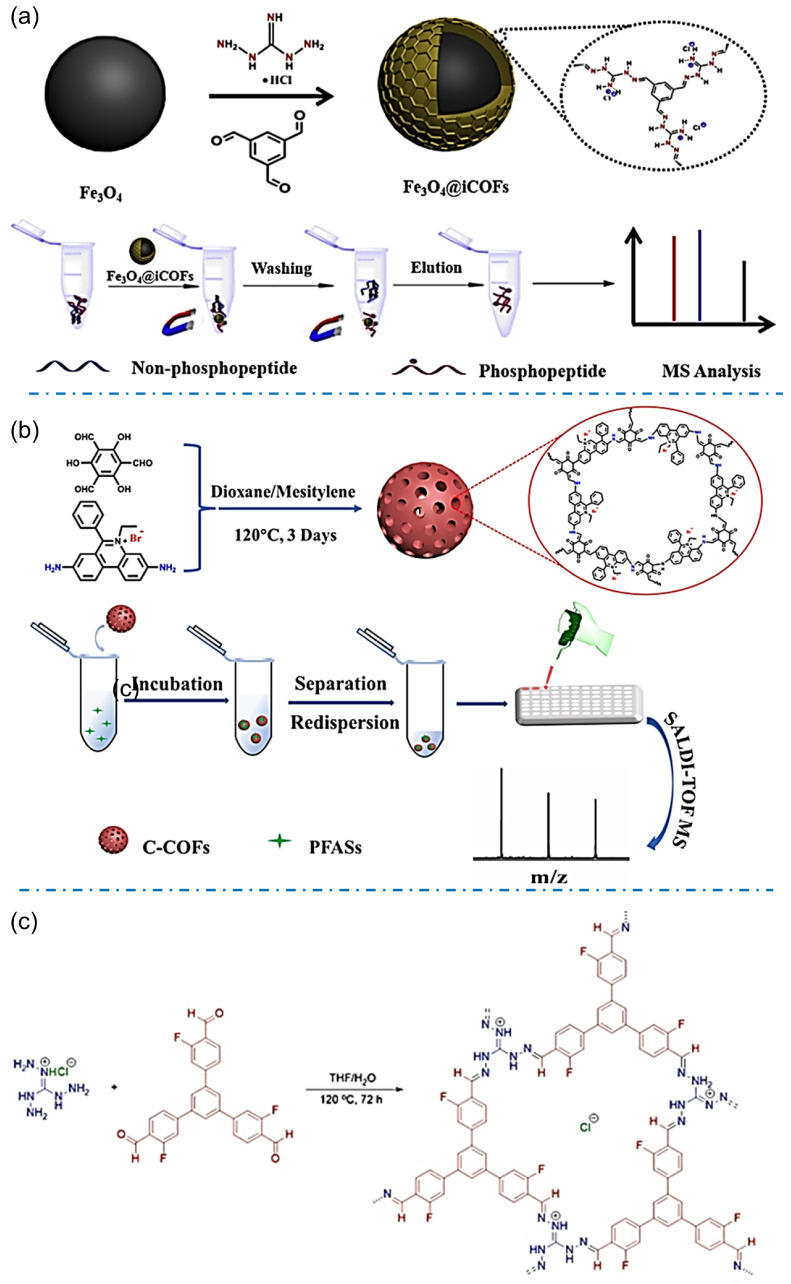
(**a**) Synthetic scheme of Fe_3_O_4_@iCOFs and typical process for selective enrichment of phosphopeptides. Reprinted with permission from [69]. Copyright 2022 Elsevier. (**b**) Schematic diagram of the synthesis principle and detection of C-COFs. Reprinted with permission from [70]. Copyright 2022 Elsevier. (**c**) Synthesis of F-iCOFs and their application in MALDI-MS detection. Reprinted with permission from [45]. Copyright 2022 Springer Nature.

Designable structures of iCOFs provide more choices to specifically enrich molecules from targeting bionic or environmental samples. The iCOFs based on guanidinium linkages are widely used in enrichment or extraction for coupling detection. For example, Xiong et al. synthesized the magnetic Fe_3_O_4_@iCOFs with guanidinium-based ionic ligands and used them for the detection of phosphopeptides (Figure 9a) [69]. The enrichment of phosphopeptides was achieved based on electrostatic interactions and hydrogen bonding between phosphate groups and guanidine groups. The combination with mass spectrometric detection gives high sensitivity and the lowest detection limit of 0.4 fmol for phosphopeptides detection in complex samples. It opens up a new avenue for phosphoproteome analysis and other biomedical applications.

The analyte-induced iCOF design provides a promising strategy for solving some environmental or healthy problems. The typical example is that iCOF with adjustable pore structures has been considered the best candidate to extract and detect per- and polyfluoroalkyl substances (PFAS) or other F-containing substances. Zhao’s group reported a magnetic ionic covalent organic backbone (Fe_3_O_4_@EB-iCOFs) that demonstrated excellent potential for magnetic solid-phase extraction of perfluorinated compounds (PFCs) due to its high surface area, partial ionic properties, and magnetic responsiveness [62]. Good linearity in the range of 1~1000 ng/L (*r* ≥ 0.995) was obtained with good reproducibility and a lower detection limit (0.1~0.8 ng/L).

Due to the extremely low content of PFASs in complex samples, an effective and simple pretreatment is necessary for the detection of trace or even ultra-trace PFASs in real samples. Sun et al. prepared ionic covalent organic skeletons (TPB-BFBIm-iCOF) with controlled size, high crystallinity, and abundant active adsorption sites for PFASs enrichment through electrostatic interactions, hydrophobic effect, and ordered channel structures [47]. Under optimal conditions, the sensitive and rapid analysis of PFASs was performed in five complex seafood media with lower detection limits (≤0.0017 ng/g). A cationic-graded porous covalent organic backbone (C-H-COF) prepared by Ye and coworkers also showed good enrichment selectivity for PFASs due to the dual effect of internal adsorption sites and electrostatic interactions [50]. The hierarchical porous structure of C-H-COF may accelerate the mass transfer of analytes, allowing the analytical detection to be completed within 10 min with improved sensitivity and a detection limit of 0.01~0.29 ng/L by using matrix-assisted laser desorption/ionization time-of-flight mass spectrometry (MALDI-TOF MS). Tang et al. [70] synthesized well-compatible and stable cationic COFs (C-COFs) for the detection of perfluoroalkyl sulfonates (PFASs) coupling with the mass spectrometric method (Figure 9b). C-COFs can rapidly and efficiently enrich PFASs such as perfluorobutylenesulfonic acid (PFBS), perfluorohexanesulfonic acid (PFHxS), and perfluorooctylsulfonic acid (PFOS) through hydrophobic, electrostatic and anion exchange interactions. The detection limits for PFBS, PFHxS, and PFOS were 0.001, 0.01, and 0.3 ng/mL, respectively based on the enrichment by using C-COFs. Liu et al. prepared a fluorine-functionalized iCOF (F-iCOF, Figure 9c) [45]. Benefiting from the presence of fluorine atoms and guanidine units, F-iCOFs not only maintain the properties of conventional COFs, but also have strong electrostatic attraction, F-F interaction, and hydrogen bonding between F-iCOFs and the target compounds, and thus reveal high selectivity and high enrichment capacity for perfluorinated sulfonate (PFS) contaminants. Furthermore, potassium perfluorobutane sulfonate (PFBSK) was detected in whole blood with a detection limit as low as 0.04 pg/mL comparable to or better than existing methods for the detection of PFSs.

### 3.2. Sensing Applications

Sensing is one of the most commonly used tools in modern chemical analysis. As a new generation of porous materials, iCOFs have the inherent advantages of predictable structures, tunable functionalities high porosity, and abundant charged sites apart from their adjustable pore size. Their amazing response to electrochemical, photo-irradiating, and electric stimulus promises iCOFs as excellent transducers for direct sensing and detection. Based on the structural design, iCOFs have been extensively applied in biosensing, environmental molecule sensing, ion detection, and so on.

#### 3.2.1. Biosensing

**Figure 10:**
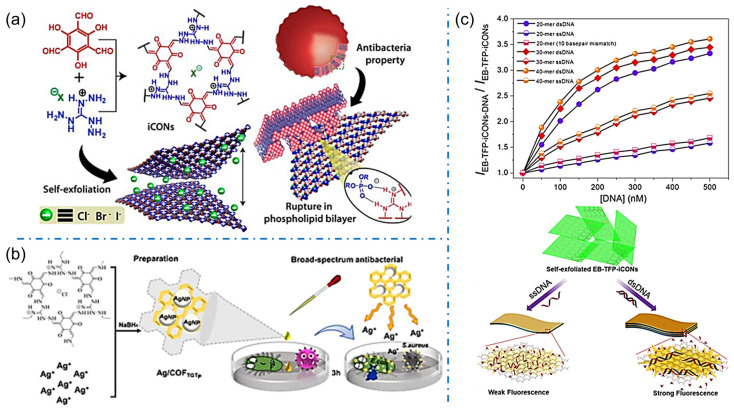
(**a**) Schematic representation of the exfoliated iCONs with bioactivity. Reprinted with permission from [40]. Copyright 2016 American Chemical Society. (**b**) Schematic diagram of the formation of Ag/COF_TGTp_ and its bioactivity. Reprinted with permission from [51]. Copyright 2022 Elsevier. (**c**) Schematic diagram of label-free detection of dsDNA by using EB-TFP-iCOFs. Reprinted with permission from [72]. Copyright 2018 Wiley-VCH.

By modulating the porous structure and charged nature, iCOFs can be used for biosensing with high sensitivity and good biocompatibility. The self-exfoliation of iCOFs was primarily investigated to achieve ionic covalent organic nanosheets (iCONs) due to the intrinsic ionic electrostatic repulsion between the organic layers. Given the stable and abundant self-exfoliating nanosheets from iCOFs, iCONs can be homogeneously dispersed in solutions for bioactive applications. In 2016, Banerjee’s group synthesized three self-exfoliating halogenated guanidinium-based iCONs (TpTG_X_, Figure 10a) [40]. The synthesized TpTG_X_ showed good antibacterial activity against Gram-positive (*S. aureus*) and Gram-negative bacteria (*E. coli*) ascribed to electrostatic interactions between the positively charged molecules of bacterial membranes or negatively charged phospholipid bilayers and iCONs. A propidium iodide ion covalent organic backbone (PI-TFP) using cucurbit[7]uril (CB[7]) was reported to induce molecular recognition in 2020 [46]. The surface charge of self-exfoliated PI-TFP was used to control the growth of *E. coli* and *S. aureus* bacteria.

Apart from the iCONs, iCOF-based materials were also proposed for bioactive sensing applications. An electrochemical impedance spectra (ESI) biosensor was proposed by Valant et al. based on a new cationic covalent organic polymer (CATN) consisting of porphyrin and pyridine units which can interact with negatively charged bacteria through electrostatic interactions [71]. The EIS biosensor exhibited a consistent response within 5 min for *E. coli* bacteria with a detection limit as low as 2 CFU/mL. Zhang et al. synthesized an antibacterial 2D COF (COF_TGTp_) containing guanidinium-based cations and used it as a carrier to form Ag/COF_TGTp_ nanocomposites (Figure 10b) [51]. By disrupting the bacterial cell wall and killing the bacteria with low cytotoxicity and hemolytic properties, Ag/COF_TGTp_ nanocomposites are expected to be new broad-spectrum and efficient antibacterial materials. In the inhibition experiments, the lowest inhibitory concentrations detected by Ag/COF_TGTp_ were 100 μg/mL and 50 μg/mL for *E. coli* and *S. aureus*, respectively. The sensitive detection of bacteria and antibacterial performance of iCOFs shed light on the advanced technologies to address the issues related to environmental conditions and human health.

Furthermore, iCONs can also be used in sensing various charged biomolecules such as DNA, amino acids, proteins, etc. via their strong and specific affinity. For example, Mal et al. designed and synthesized the self-exfoliated crystalline 2D EB-TFP-iCONs in an aqueous medium with brilliant fluorescence at 600 nm (Figure 10c) [72]. The EB-TFP-iCONs can reassemble in the presence of dsDNA forming EB-TFP-iCONs-DNA hybrids. The recombination is highly selective for dsDNA compared to single-stranded (ssDNA), which allows EB-TFP-iCONs to act as the 2D fluorescent probe for label-free detection of complementary DNA strands. Overall, the use of iCOF in biosensing has promising applications in the fields of medicine, biology, and environmental monitoring. Further research and development of iCOF-based biosensors can lead to the development of more efficient and accurate detection methods.

#### 3.2.2. Molecule Sensing

With the tailored structures and charged properties, iCOFs can be used to directly recognize and detect a range of molecules including pollutants, antibacterial, and specific molecules with high selectivity and sensitivity, promising iCOFs as potential materials for environmental monitoring, human health, and food security. 

**Figure 11:**
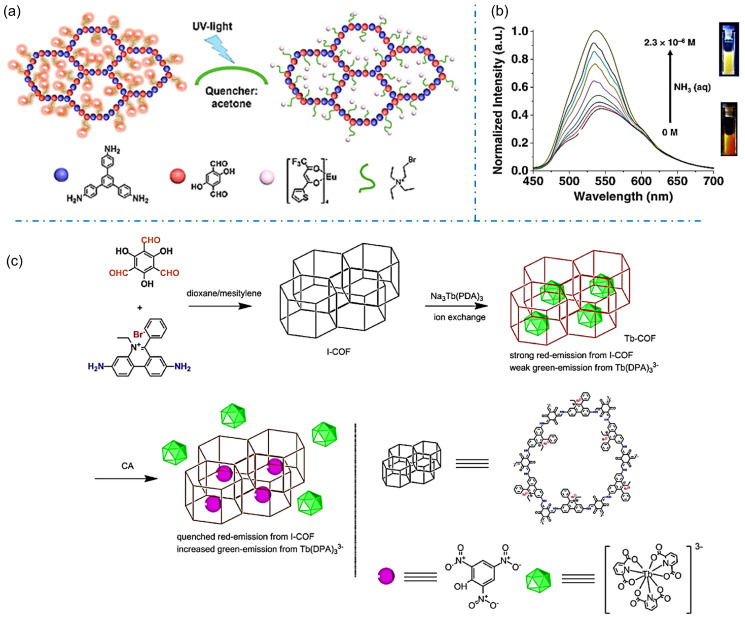
(**a**) Schematic diagram of the fluorescence-quenched detection of acetone based on DhaTab-COF-EuIL. Reprinted with permission from [52]. Copyright 2019 American Chemical Society. (**b**) Amine response behavior of TGH^+^⸱PD [43]. (**c**) Synthesis of Tb-COF for target molecule detection. Reprinted with permission from [75]. Copyright 2022 Elsevier.

The selective uptake of the guest molecules into iCOFs can lead to significant changes in luminescence, making them excellent candidates for chemical sensors. In 2019, Zuo et al. reported a Europium (Eu)-grafted COF (DhaTab-COF-EuIL) by ionic liquid (IL) modification and ion replacement based on the chemically stable covalent organic backbone (DhaTab-COF, Figure 11a) [52]. The fluorescence of DhaTab-COF-EuIL could be quenched in the presence of acetone. High sensitivity and selectivity for detecting volatile acetone were obtained with the detection limit down to 1%. Given that acetone is also a common metabolite in the human body, the sensitive DhaTab-COF-EuIL-based fluorescent sensor could be applied in the non-invasive diagnosis of diabetes, renal function, and the ketogenic diet. Das et al. designed an iCOF (TGH^+^⸱PD) consisting of guanidine and phenanthroline fractions for the rapid fluorescent detection of ammonia and aliphatic amines through a turn-on strategy (Figure 11b) [43]. An ultra-low detection limit for ammonia was 1.2 × 10^−7^ M, which endows TGH^+^⸱PD as an effective material for monitoring the freshness of meat products. These works provide new design ideas for fluorescent signal molecular recognition systems. 

The sensitive detection of deleterious chemicals or pollutants in the environment is another important application of iCOFs. In 2022, Xu et al. synthesized an ionic Tb-COF by loading charged rare-earth Tb(III) (Tb(DPA)_3_^3−^, DPA = pyridine-2,6-dicarboxylic acid) as a luminescent emitter into the hexagonal I-COF cage modified with an ionic exchange ratio of 41% (Figure 11c) [73]. The synthesized Tb-COF showed weak green Tb(III) emission and strong red I-COF emission. The addition of picric acid (PA), a widely used but hardly degraded molecule in the environment, induced the increased Tb(III) emission whereas weakened I-COF emission. The ratiometric sensing behavior was ascribed to the energy competing on Tb(III) ^5^D_4_ level as the “dopant replacement” effect in the presence of PA. By using the same strategy, Zhu et al. reported two Tb(III) ions doped C-COF (TbDBM-COF and TbCF_3_-COF) by ion-exchange reaction as probes for PA sensing [74]. A linear response was found in the PA concentration region of 0–9 μM. TbCF_3_-COF demonstrated better sensing performance with a lower detection limit of 0.9 μM due to its suitable ligand triplet energy level. Sun et al. reported the Tb(DPA)_3_^3−^-loaded I-COF (Tb-COF) for the sensing and removal of chrysolepic acid (CA) molecules (Figure 11c) [75]. The Tb-COF probe was able to generate a proportional emission signal corresponding to the concentration of CA in the range of 0–9 μM. These works indicate the potential applications of designed iCOF-based fluorescent probes and molecular recognition systems in effectively addressing environmental issues.

#### 3.2.3. Ion Sensing

**Figure 12:**
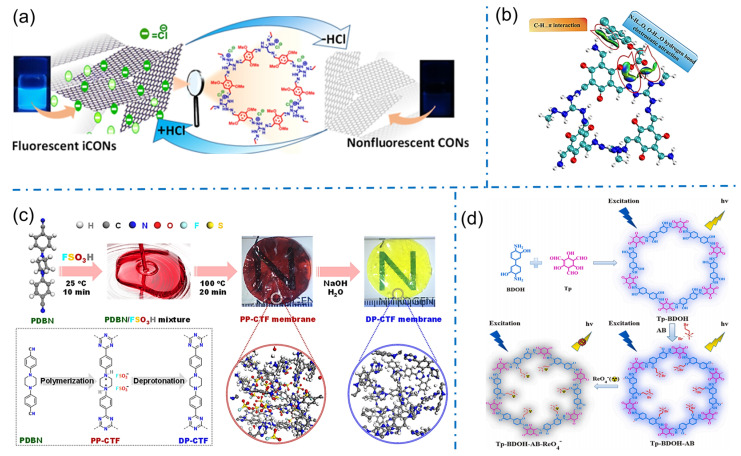
(**a**) Schematic diagram of the proton-triggered fluorescence switching of DATG_Cl_-iCONs for fluorine detection. Reprinted with permission from [44]. Copyright 2020 American Chemical Society. (**b**) The interaction of the analyte with TpTG_Cl_. Reprinted with permission from [41]. Copyright 2022 Elsevier. (**c**) Chemical illustration of synthetic PP-CTF and DP-CTF membranes. Reprinted with permission from [76]. Copyright 2021 American Chemical Society. (**d**) Schematic diagram of the synthesis of Tp-BDOH-AB and its application in selective detection of ReO_4_^−^. Reprinted with permission from [53]. Copyright 2022 Elsevier.

Benefiting from their unique properties, iCOFs also show tremendous potential in ion-sensing applications. Particularly, iCOFs can be tailored to selectively detect fluoride or other anionic ions in various environmental and biological samples. In 2020, Pal’s team reported the first case of fluorine ion sensing based on a self-exfoliated fluorescent DATG_Cl_-iCONs consisting of appropriate fluorophores and triaminoguanidinium chloride (TG_Cl_) as a cationic junction (Figure 12a) [44]. Due to its cationic nature and well-exposed active sites, DATG_Cl_-iCONs could selectively detect fluorine (F^−^) ions with proton triggering even at a concentration as low as 5 ppb. The F^−^ ion recognition was also indicated by the naked eye for fast response and recovery due to the visible fluorescent signal of DATG_Cl_-iCONs. This work provides a fundamental pathway for the development of photoluminescent artificial molecular switches based on 2D organic nanosheets. In 2022, Liu et al. prepared a similar TG_Cl_-based iCOF (TpTG_Cl_) with a large number of positive charges and hydrogen bonding sites for faster and more sensitive detection of anionic phenoxy carboxylic acids (PCAs) through the fluorescent method (Figure 12b) [41]. The strong electrostatic adsorption of TpTG_Cl_ to PCAs was successfully applied to the quantitative analysis of five PCAs in rice and water samples with the lowest detection limits of 0.016–0.036 ng/g for rice samples and 0.43–0.78 ng/L for water samples.

The modified iCOFs from non-ionic COFs were also developed for ion sensing. Yang et al. prepared robust covalent triazine framework (CTF) membranes with ionic functionality by a super-strength acid (FSO_3_H) for indicating pH alternation (Figure 12c) [76]. The ion pairs of FSO_3_^−^ anions and ammonium cations were formed within the protonated PP-CTF backbone by introducing piperazine groups as reaction sites. The transparent red protonated PP-CTF membranes demonstrated remarkable and stable changes in optical absorption and emission behavior as deprotonated under alkaline conditions, acting as a pH indicator. In 2022, Yi et al. reported the efficient fluorescent detection of ReO_4_^−^ by using an ionic liquid-modified COF (Tp-BDOH-AB) with unique structural and compositional features (Figure 12d) [53]. Immobilized ionic liquids on the channel walls of COF and exposed binding sites of positive layers increased the affinity between the backbone and ReO_4_^−^, conferring a stronger binding drive. The regular and hydrophobic pore channels of Tp-BDOH-AB accelerated the diffusion kinetics of ReO_4_^−^ and bonded the anions with high charge density. These designed structural features resulted in an ultra-fast fluorescent response (2 s) to ReO_4_^−^ with a detection limit as low as 1.04 μM. This work demonstrates for the first time the great potential of iCOFs for the selective detection of ReO_4_^−^ and provides an opportunity for the detection and removal of ReO_4_^−^ in the real environment.

#### 3.2.4. Others

In addition to the above applications, iCOF’s unique properties also make it a promising material for humidity and temperature sensing. In 2020, Zhang et al. reported a humidity sensor by using a β-ketoamine linked iCOF (TpPa-SO_3_Na) [55]. The unique layered structure, fixed pore size, and abundant active sites (-SO_3_Na, -NH) of TpPa-SO_3_Na allow easy adsorption, migration, and sensing of water molecules in the pores. The following year, Xian et al. proposed a temperature sensor based on an iCOF nanofluidic membrane [56]. By separating two electrolyte solutions through a membrane, the temperature difference on the membrane can synchronize the induced potential based on thermally driven ion charge separation. Using these properties, it has promising applications in environmental temperature monitoring. This temperature sensor fills the gap of current temperature response materials.

## 4. Conclusions and Perspectives

In summary, iCOFs have drawn extensive attention in chemical analysis through rational structure design and construction in recent years. The charged frameworks or pores coupling with the inherited characteristics of COFs make iCOFs attractive for chemically analytical applications. The adjustable well-defined pore channel and electrostatic interaction are desirable to extract ions or molecules with opposite charges, further enriching these matters in the pore channels with high specific surface area and fast mass transport dynamics. The tunable molecular structures and abundant interaction sites of iCOFs promise the ability to recognize the target analytes with accurate selectivity and high sensitivity. Furthermore, the intrinsic multi-functionalities of iCOFs also contribute to the analytical transduce performance for biosensing, environmental analysis, surroundings monitoring, etc. The brilliant characteristics of iCOFs coupled with the advanced analytical methodologies feature the potential applications of iCOFs in chemical analysis.

Since the first report on iCOF synthesis in 2013, more and more fabrication strategies, physicochemical properties, and extensive applications of iCOFs have been investigated after 2015. Tremendous progress has been made in just a few years. The iCOFs are demonstrating great potential in the field of chemical analysis. However, the iCOF-based techniques for chemically analytical applications are still in their beginning stages. There are still several challenges facing the development of iCOF-based analytical methods. Firstly, the ionic monomers for constructing iCOFs are still limited, mainly focusing on bipyridinium (BIPY^2+^), EB, halogenated guanidinium (TG_x_), ionic liquids (ILs), etc. Only a few anionic and zwitterionic COFs have been developed and applied in chemical analysis. Exploring novel ionized molecules for iCOF design and fabrication is an attractive direction. Secondly, many efforts are desired to be made to achieve the required properties for analytical detection by optimizing the structure design, synthetic methods, and characterization of iCOF-based materials. The relationship between the structure and the performance should be illustrated in future works. Thirdly, developing iCOF-based sensors requires a thorough understanding of their interactions with biomolecules. Last but not least, sensing applications of iCOFs as transducers are dominated by the fluorescent method. Only a few electrochemical and electric sensors have been reported to date. The more convenient, fast, sensitive, and accurate sensing platforms or devices are desirable for the next iCOF-based analytic applications.

By addressing the present challenges facing iCOF-based analytical applications, it is attractive to develop more accurate, sensitive, and reliable analytical methodologies for greater contribution to environmental monitoring, food security, biomedicines, and human health.

## Figures and Tables

**Table 1:** Structural parameters of typical iCOFs reported in previous works.

Entry	iCOF Name	Type	Monomer	Charged Methods	Pore Size (nm)	Surface Area (m^2^/g)	Ref.
**1**	3D-ionic-COF	Cationic COF	**21+26**	Initial charged	0.86	966	[25]
2	EB-COF:Br	Cationic COF	**13+26**	Initial charged	1.66	774	[34]
3	PC-COF	Cationic COF	**1+23**	Initial charged	5.8	n/a ^a^	[35]
4	cCTFs-500	Cationic COF	**25**	Initial charged	1.64	1247	[39]
5	TpTG_X_	Cationic COF	**13+32**	Initial charged	1.2	267	[40]
6	TpTG_Cl_	Cationic COF	**13+32**	Initial charged	3.48	167	[41]
7	BT-DG_Cl_	Cationic COF	**15+32**	Initial charged	n/a	3 ^b^	[42]
8	TGH^+^⸱PD	Cationic COF	**17+32**	Initial charged	n/a	16 ^b^	[43]
9	DATG_Cl_-iCONs	Cationic COF	**18+32**	Initial charged	n/a	155	[44]
10	F-iCOF	Cationic COF	**20+32**	Initial charged	2.5	148	[45]
11	PI-TFP-iCONs	Cationic COF	**13+27**	Initial charged	1.57	535	[46]
12	TPB-BFBIm-iCOF	Cationic COF	**1+29**	Initial charged	4.45	182.83~189.51	[47]
13	Co-TpBpy	Cationic COF	**7+13**	Post-modification	2.1	450	[48]
14	Py-BPy^2+^-COF	Cationic COF	**2+16**	Post-modification	2.1	461	[49]
15	C-H-COF	Cationic COF	**7+13**	Post-modification	n/a	711.6	[50]
16	COF_TGTp_	Cationic COF	**11+13**	Post-modification	3.4	81.69	[51]
17	DhaTab-COF-EuIL	Cationic COF	**1+19**	Post-modification	3.7	2061	[52]
18	Tp-BDOH-AB	Cationic COF	**8+13**	Post-modification	1.4	140	[53]
19	TpPa-SO_3_Li	Anionic COF	**3+13**	Deprotonation	1.18	348	[36]
20	TpPa-SO_3_Na	Anionic COF	**4** **+13**	Deprotonation	1.40	212.3	[54,55]
21	COF-COOH	Anionic COF	**10+13**	Deprotonation	1.2	399	[56]
22	H-Li-ImCOF	Anionic COF	**9+15**	Post-modification	2.9	350	[57]
23	CD-COFs	Anionic COF	**/**	Initial charged	0.64	760	[58]
24	CuP-SQ COF	Zwitterionic COF	**31**	Initial charged	2.1	539	[59]
25	XJCOF	Zwitterionic COF	**13+4/26**	Initial charged	1.5/1.7	467~503	[60]
26	[BE]_X%_-TD-COFs	Zwitterionic COF	**1+19**	Post-modification	3.24~2.95	2020~470	[38]

Notes: a: The n/a represented that the corresponding pore size and specific surface area were not presented in the reported works. b: The surface areas of BT-DG_Cl_ and TGH^+^⸱PD were pretty low, which was attributed to their poor crystallinity.

**Table 2:** Performances of iCOF-based analytical methods and sensors.

Entry	iCOF Name	Analytical Methods ^a^	Analytes	Limit of Detection ^b^	Ref.
1	PS-IL-COFs	Enrichment/extraction + MS	propofol	0.18 μg/L	[67]
2	Fe_3_O_4_@EB-TFB-iCOF	Enrichment/extraction + MS	organic acid	0.1~0.49 ng/mL	[68]
3	Fe_3_O_4_@iCOFs	Enrichment/extraction + MS	phosphopeptides	0.4 fmol	[69]
4	Fe_3_O_4_@EB-iCOFs	Enrichment/extraction + MS	perfluorinated compounds	0.1~0.8 ng/L	[62]
5	TPB-BFBIm-iCOF	Enrichment/extraction + MS	PFASs	≤0.0017 ng/g	[47]
6	C-H-COF	Enrichment/extraction + MS	PFASs	0.01~0.29 ng/L	[50]
7	C-COF	Enrichment/extraction + MS	PFBS/PFHxS/PFOS	0.001/0.01/0.3 ng/mL	[70]
8	F-iCOF	Enrichment/extraction + MS	PFBSK	0.04 pg/mL	[45]
9	CATN	EIS	*E. coli*	2 CFU/mL	[71]
10	Ag/COF_TGTp_	Staining experiment	*E. coli* & *S. aureus*	100/50 μg/mL	[51]
11	EB-TFP-iCONs	Fluorescent sensing	dsDNA	n/a	[72]
12	DhaTab-COF-EuIL	Fluorescent sensing	acetone	1%	[52]
13	TGH^+^⸱PD	Fluorescent sensing	ammonia	1.2 × 10^−7^ M	[43]
14	Tb-COF	Fluorescent sensing	picric acid (PA)	n/a	[73]
15	TbCF_3_-COF	Fluorescent sensing	PA	0.9 μM	[74]
16	Tb-COF	Fluorescent sensing	chrysolepic acid (CA)	0~9 μM	[75]
17	DATG_Cl_-iCONs	Fluorescent sensing	F^−^	5 ppb	[44]
18	TpTG_Cl_	Fluorescent sensing	phenoxy carboxylic acids (PCAs)	0.016~0.036 ng/g	[41]
19	PP-CTF	Fluorescent sensing	H^+^	n/a	[76]
20	Tp-BDOH-AB	Fluorescent sensing	ReO_4_^−^	1.04 μM	[53]
21	TpPa-SO_3_Na	Electrical testing	Humidity	n/a	[55]
22	COF-COOH	Electrical testing	Temperature	n/a	[56]

Notes: a: The MS represented mass spectrometry. EIS was the abbreviation of electrochemical impedance spectroscopy. b: The n/a meant that there was no limit of detection provided in the works.

## Data Availability

Not applicable.
